# Early post-endovascular treatment contrast extravasation on dual-energy CT is associated with clinical and radiological stroke outcomes: A 10-year single-centre experience

**DOI:** 10.1177/23969873231157901

**Published:** 2023-02-22

**Authors:** Florentina ME Pinckaers, Max MG Mentink, Hieronymus D Boogaarts, Wim H van Zwam, Robert J van Oostenbrugge, Alida A Postma

**Affiliations:** 1 Department of Radiology and Nuclear Medicine, Maastricht University Medical Centre, Maastricht, The Netherlands; 2 School for Cardiovascular Diseases (CARIM), Maastricht University, Maastricht, The Netherlands; 3 Department of Neurosurgery, Radboud UMC, Nijmegen, The Netherlands; 4 Department of Neurology, Maastricht University Medical Centre, Maastricht, The Netherlands; 5 School for Mental Health and Neuroscience (MHENS), Maastricht University, Maastricht, The Netherlands

**Keywords:** Dual-energy CT, contrast extravasation, iodine, endovascular treatment, ischaemic stroke, treatment outcome

## Abstract

**Objective:**

To determine the association between early post-endovascular treatment (EVT) contrast extravasation (CE) on dual-energy CT (DECT) and stroke outcomes.

**Methods:**

EVT records in 2010–2019 were screened. Exclusion criteria included the occurrence of immediate post-procedural intracranial haemorrhage (ICH). Hyperdense areas on iodine overlay maps were scored according to the Alberta Stroke Programme Early CT Score (ASPECTS), thus forming a CE-ASPECTS. Maximum parenchymal iodine concentration and maximum iodine concentration relative to the torcula were recorded. Follow-up imaging was reviewed for ICH. The primary outcome measure was the modified Rankin Scale (mRS) at 90 days.

**Results:**

Out of 651 records, 402 patients were included. CE was found in 318 patients (79%). Thirty-five patients developed ICH on follow-up imaging. Fourteen ICHs were symptomatic. Stroke progression occurred in 59 patients. Multivariable regression showed a significant association between decreasing CE-ASPECTS and the mRS at 90 days (adjusted (a)cOR: 1.10, 95% CI: 1.03–1.18), NIHSS at 24–48 h (aβ: 0.57, 95% CI: 0.29–0.84), stroke progression (aOR: 1.14, 95% CI: 1.03–1.26) and ICH (aOR: 1.21, 95% CI: 1.06–1.39), but not symptomatic ICH (aOR 1.19, 95% CI: 0.95–1.38). Iodine concentration was significantly associated with the mRS (acOR: 1.18, 95% CI: 1.06–1.32), NIHSS (aβ: 0.68, 95% CI: 0.30–1.06), ICH (aOR: 1.37, 95% CI: 1.04–1.81) and symptomatic ICH (aOR: 1.19, 95% CI: 1.02–1.38), but not stroke progression (aOR: 0.99, 95% CI: 0.86–1.15). Results of the analyses with relative iodine concentration were similar and did not improve prediction.

**Conclusions:**

CE-ASPECTS and iodine concentration are both associated with short- and long-term stroke outcomes. CE-ASPECTS is likely a better predictor for stroke progression.

## Introduction

The beneficial effect of endovascular treatment (EVT) on patient outcomes after acute ischaemic stroke (AIS) in the anterior circulation has been well established and has led to its implementation as standard care.^1^ On post-EVT CT imaging, the brain parenchyma often shows high-attenuation areas.^2^ With dual-energy CT (DECT), it is possible to distinguish whether this is caused by intracranial haemorrhage (ICH), or the extravasation of iodinated contrast due to blood-brain barrier dysfunction (BBBD; Supplemental Appendix I).^2,3,4^

BBBD has been linked to both reperfusion injury and haemorrhagic transformation.^5^ Similarly, several studies have described a relationship between early contrast extravasation (CE) and haemorrhagic complications or poor clinical outcome.^6–9^ However, previous literature has mainly focussed on either the absence versus presence of CE,^6,7^ or on the maximum iodine concentration in a specific ROI.^8,9^ Either measure may not best reflect the total extent of ischaemic damage, and could therefore be less suitable to predict short- and long-term outcomes. Furthermore, previous studies had relatively small sample sizes, which may have limited their capability to detect clinically relevant effects.

With this single-centre study, we aim to examine the association between early post-EVT CE on DECT and clinical and radiological outcomes after AIS in the anterior circulation. This research question will be explored with an Alberta Stroke Programme Early CT Score (ASPECTS)-based score, and with iodine concentration measures.^8–10^

## Methods

### Patient selection

EVT records of the Maastricht University Medical Centre (MUMC) from 2010 to 2019 were screened. Patients with an occlusion of the internal carotid artery (ICA), ICA-terminus (ICA-T) or middle (M1/M2) cerebral artery were included. Participation in the MR CLEAN LATE trial was an exclusion criterion because of the trial’s blinded endpoint setting.^11^ MR CLEAN MED patients who received aspirin or heparin were excluded, as this trial medication may have affected their ICH risk.^12^ Other reasons for exclusion were: (1) post-procedural DECT not performed; (2) DECT not performed within 3 h post-EVT; (3) loss of DECT source data and reconstructions. Finally, patients with ICH on post-procedural DECT were excluded from our main analysis.

### Statement of ethics approval

This study was approved by the ethics committee of the MUMC, Maastricht, The Netherlands (MEC-2020-1456). The need for individual patient consent was waived.

### Imaging protocol

As part of the standard follow-up protocol, DECT was performed immediately post-EVT on a second- or third generation dual-source CT (Somatom Definition Flash/Force, Siemens Healthcare, Forchheim Germany). A dedicated dual-energy protocol with simultaneous imaging at either (1) 80/Sn140 kVp, 392/196 mAs eff. (Flash first years; CTDI 37 mGy), (2) 80/Sn140 kVp, 500/250 mAs eff. (Flash later years; CTDI 32.3 mGy) or (3) 80/Sn150 kVp, 310/207 mAs eff (Force; CTDI 24.5 mGy) was employed, with collimation of 0.6 mm and pitch of 0.7. Sn indicates extra filtration by a tin filter.

Mixed-energy images were generated from high and low kVp images, providing a ‘conventional single-energy CT’ comparable to 120-kVp images with a weighted average of 0.6 (Flash) and 0.5 (Force). A dedicated brain haemorrhage algorithm was used to calculate virtual non-contrast images (VNC), iodine overlay maps (IOM) and iodine concentration (SyngoVia VB40, Siemens Healthcare).

### Imaging assessment

Because of their inclusion in either the MR CLEAN pretrial, MR CLEAN trial, MR CLEAN Registry, MR CLEAN NO-IV or MR CLEAN MED, core lab imaging assessments were available for most patients.^12–16^ If such assessments were not readily available, imaging was assessed by an experienced neuroradiologist and core lab member (AP).

**Figure 1.**
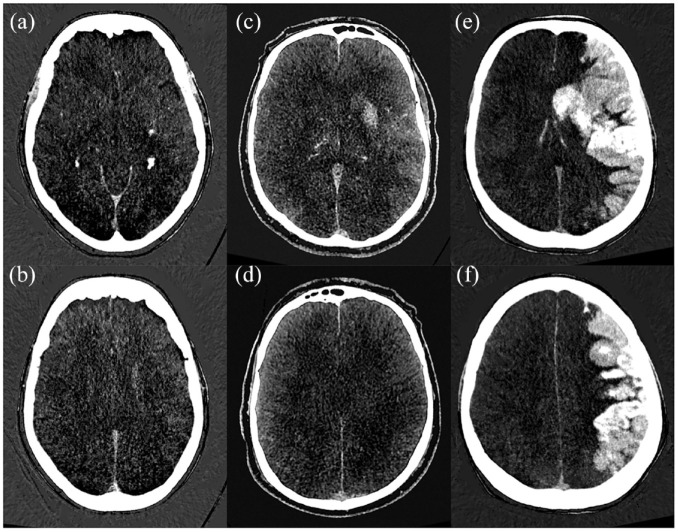
Contrast extravasation ASPECTS (CE-ASPECTS) on DECT iodine maps. In patient 1 (a and b), the caudate and lentiform nucleus show contrast extravasation (CE-ASPECTS = 8). In patient 2 (c and d), the caudate and lentiform nucleus, insula, M2 and M3 are affected (CE-ASPECTS = 5). In patient 3 (e and f), all areas but the posterior limb of the internal capsule are affected (CE-ASPECTS = 1).

Hyperdense areas on IOM were scored according to the ASPECTS by two readers in consensus (AP and FP), thus forming a CE-ASPECTS (Figure 1). One reader (FP) measured maximum paenchymal iodine concentration in the middle cerebral artery territory by placing a 0.3 cm^2^ ROI at the location of maximum visual attenuation. If no CE was observed, the ROI was placed in the head of the ipsilateral caudate nucleus. An additional 0.1 cm^2^ ROI was placed in de superior sagittal sinus (SSS) near the confluence.^9^ Maximum iodine concentration (mg/ml) and iodine concentration relative to the SSS were recorded.

VNC reconstructions of post-EVT DECT and any additional follow-up scans were evaluated for ICH according to the Heidelberg criteria by an experienced neuroradiologist with >10 years’ experience with DECT (AP).^17^ ICH evaluation took place with access to previous imaging. Patients who participated in the MR CLEAN trial, MR CLEAN NO-IV or MR CLEAN MED received follow-up imaging as part the trial protocol (*n* = 37).^12,14,16^ All other follow-up imaging was performed on clinical indication.

### Study parameters

Clinical baseline and follow-up data were gathered from prospective records. The primary outcome measure was the modified Rankin Scale (mRS) score at 90 days; secondary outcome measures were the National Institutes of Health Stroke Scale (NIHSS) score at 24–48 h, poor functional outcome at 90 days (mRS 3-6), mortality at 90 days, stroke progression, ICH and symptomatic ICH (sICH). Patients who were deceased at 24–48 h received an NIHSS score of 42. Stroke progression was defined as neurological deterioration of ≥4 points on the total NIHSS score, or ≥2 points on any NIHSS category. sICH was scored in accordance with the Heidelberg criteria.^17^

### Missing data

As the availability of DECT reconstructions was an inclusion criterium, CE-ASPECTS could be assessed in all patients. In 49 patients (12%), iodine measurements could not be performed as a result of missing source images. The percentage of missing data in the outcome variables mRS at 90 days and NIHSS at 24–48 h was 2.7% and 4.5%, respectively.

Multiple imputation by chained equations (MICE) using the mice package (version 3.14.0) was used to handle missing data.^18^ The imputation model included relevant covariates and outcomes. The number of imputations was based on the fraction of missing information.^19^

### Statistical analysis

We used crude data to report baseline characteristics and outcomes for three CE-ASPECTS categories (0–4, 5–7 and 8–10). The ASPECTS, CE-ASPECTS and NIHSS were all considered to be continuous variables. Inter-rater reliability (IRR) was assessed using a two-way random, agreement, single-measure intra-class correlation (ICC).

In order to estimate the effect of CE-ASPECTS and iodine concentration on our chosen outcome measures, we used univariate and multivariable linear, logistic and ordinal (shift) regression. Multivariable adjustments were based on univariate analyses (Supplemental Appendix II). Effect estimates are expressed per one point decrease in the CE-ASPECTS (equivalent to one additional region with CE), and per one point increase in iodine concentration.

All statistical analyses were performed in R version 4.2.2.^20^

## Results

### Participants

**Figure 2.**
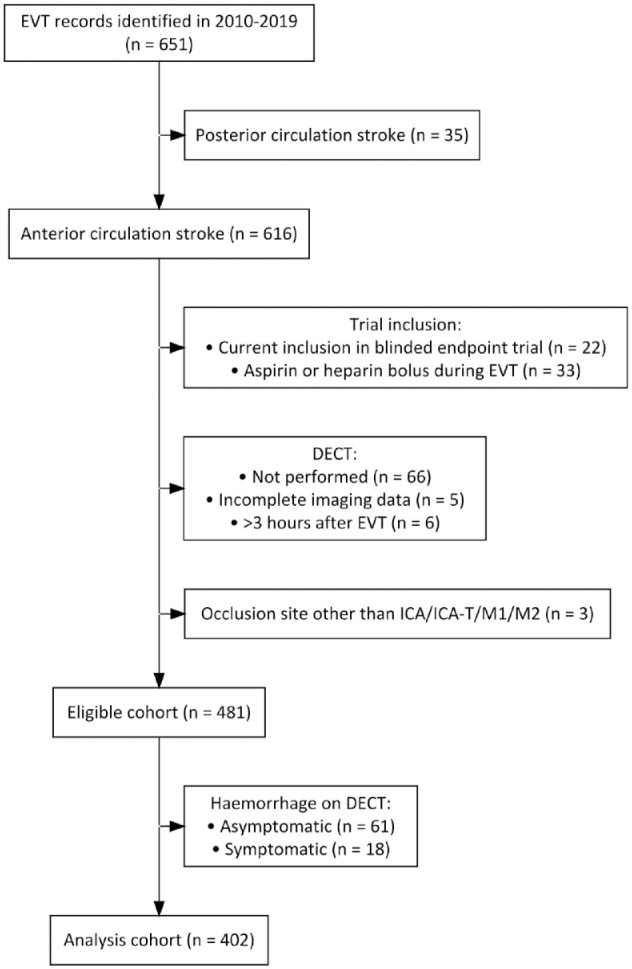
Inclusion flowchart.

We identified 651 EVT-records. Four hundred two patients met our selection criteria (Figure 2).

CE was found in 318 patients (79%). The median CE-ASPECTS was 8 (IQR 5–9). Baseline characteristics are shown in Table 1. Patients with lower CE-ASPECTS had poorer baseline ASPECTS and collateral grades (*p* = 0.01). They also had an M1 occlusion less often and had higher glucose levels (*p* = 0.04). Considering procedural characteristics, decreasing CE-ASPECTS was significantly associated with a longer EVT-duration (*p* < 0.001) and a higher number of attempts (*p* < 0.001). The ICC of CE-ASPECTS was 0.98, indicating excellent IRR.

**Table 1.** Patient characteristics.

	CE-ASPECTS 8–10 (*n* = 214)	CE-ASPECTS 5–7 (*n* = 97)	CE-ASPECTS 0–4 (*n* = 91)	*p*-value
Clinical characteristics				
Age, median (IQR)	71 (63–81)	73 (63–80)	76 (66–83)	0.18
Male sex, *n* (%)	99 (46)	48 (49)	36 (40)	0.39
NIHSS, median (IQR)	15 (10–19)	16 (11–17)	15 (10–18)	0.93
Treatment with IV alteplase, *n* (%)	165 (77)	70 (72)	65 (71)	0.25
SBP (mmHg), mean (SD)	151 (24)	150 (24)	151 (27)	0.54
DBP (mmHg), mean (SD)	81 (16)	84 (15)	81 (15)	0.83
Glucose (mmol/L), median (IQR)	6.6 (5.9–7.9)	6.8 (6.1–9.1)	7.1 (6.2–8.7)	**0.04**
Haemoglobin (mmol/L), mean (SD)	8.3 (1.1)	8.4 (1.0)	8.2 (1.1)	0.47
Medical history, *n* (%)				
Diabetes mellitus	27 (13)	15 (15)	16 (18)	0.24
Hypertension	104 (49)	47 (48)	50 (55)	0.36
Hypercholesterolaemia	56 (26)	23 (24)	21 (23)	0.53
Atrial fibrillation	51 (24)	20 (21)	20 (22)	0.64
Myocardial infarction	34 (16)	9 (9)	12 (13)	0.36
Peripheral arterial disease	13 (6)	10 (10)	8 (9)	0.30
Previous ICH	2 (1)	3 (3)	0 (0)	0.80
Previous ischaemic stroke	26 (12)	12 (12)	15 (16)	0.35
Pre-stroke mRS >2	31 (14)	14 (14)	18 (20)	0.29
Medication and intoxications, *n* (%)				
Antiplatelet	71 (33)	31 (32)	31 (34)	0.93
DOAC	8 (4)	1 (1)	9 (10)	0.05
Heparin	6 (3)	3 (3)	2 (2)	0.81
Coumarin	16 (7)	11 (11)	7 (8)	0.76
Smoking	46 (28)	29 (39)	18 (28)	0.69
Imaging characteristics				
Level of occlusion, *n* (%)				
ICA	16 (7)	6 (6)	10 (11)	0.39
ICA-T	34 (16)	19 (20)	16 (18)	0.61
M1	128 (60)	60 (62)	41 (45)	**0.04**
M2	36 (17)	12 (12)	24 (26)	0.11
ASPECTS, median (IQR)	9 (8–10)	9 (7–10)	9 (7–10)	**0.01**
Collaterals grade 2–3, *n* (%)	149 (71)	57 (61)	51 (57)	**0.01**
EVT characteristics				
Onset to groin puncture (min), median (IQR)	190 (156–270)	210 (158–285)	194 (146–249)	0.83
EVT duration (min), median (IQR)	45 (25–72)	63 (35–90)	60 (39–85)	<**0.001**
Recanalisation, *n* (%)	154 (73)	70 (73)	65 (71)	0.80
Total attempts, median (IQR)	1 (1–2)	2 (1–3)	2 (1–4)	<**0.001**
Intra-arterial heparin, *n* (%)	42 (22)	23 (24)	9 (11)	0.07
ICA stent, *n* (%)	17 (8)	13 (13)	11 (12)	0.19
Procedural complications, *n* (%)	43 (22)	31 (34)	26 (30)	0.07
Dissection	7 (4)	4 (4)	3 (3)	0.96
Perforation	0 (0)	0 (0)	0 (0)	-
Embolus new territory	8 (4)	8 (9)	8 (9)	0.07
Distal thrombus	24 (12)	20 (22)	18 (21)	**0.03**
Vasospasm	17 (9)	9 (10)	3 (3)	0.20

ASPECTS: Alberta Stroke Programme Early CT score; CE-ASPECTS: contrast extravasation ASPECTS; DBP: diastolic blood pressure; DOAC: direct oral anticoagulant; EVT: endovascular therapy; ICA: internal carotid artery; ICA-T: ICA-terminus; ICH: intracranial haemorrhage; IV: intravenous; mmHg: millimetre of mercury; mRS: modified Rankin Scale; M1/M2: middle cerebral artery; NIHSS: National Institutes of Health Stroke Scale; SBP: systolic blood pressure.

Statistically significant estimates are bold. The Cochran-Armitage test was used for nominal dependent variables, Kendall’s tau for continuous variables.

The median iodine concentration was 1.1 mg/ml (IQR 0.6–1.8). The median iodine concentration relative to the SSS was 0.9 mg/ml (IQR 0.5–1.6).

### Association with clinical outcomes

**Figure 3.**
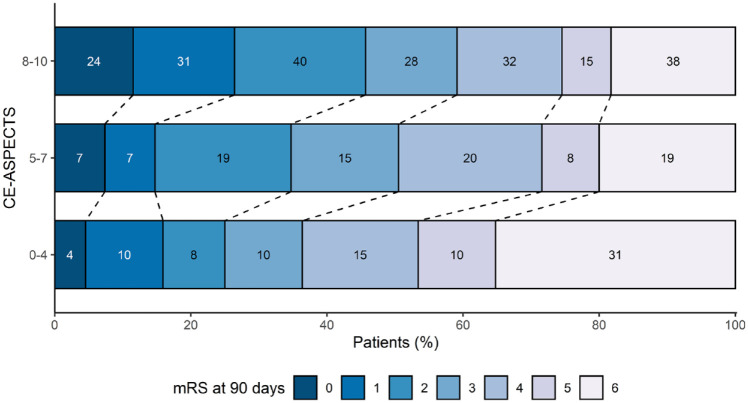
Modified Rankin Scale (mRS) distribution per contrast extravasation ASPECTS (CE-ASPECTS) category.

Clinical outcomes per CE-ASPECTS category are shown in Table 2. Decreasing CE-ASPECTS was associated with the mRS at 90 days, NIHSS score at 24–48 h and mortality at 90 days (Table 3). Figure 3 demonstrates a shift in mRS scores with decreasing CE-ASPECTS and an increased incidence of poor outcome and death.

**Table 2.** Stroke outcomes per CE-ASPECTS category.

Outcome measures	CE-ASPECTS 8–10 (*n* = 214)	CE-ASPECTS 5–7 (*n* = 97)	CE-ASPECTS 0–4 (*n* = 91)
NIHSS at 24–48 h, median (IQR)	7 (2–16)	13 (6–18)	14 (8–19)
mRS 3–6 at 90 days, *n* (%)	113 (54)	62 (65)	66 (75)
Mortality at 90 days, *n* (%)	38 (18)	19 (20)	31 (35)
Stroke progression, *n* (%)	23 (11)	13 (13)	23 (25)
ICH, *n* (%)	9 (4)	12 (12)	14 (15)
Symptomatic ICH, *n* (%)	5 (2)	3 (3)	6 (7)

ASPECTS: Alberta Stroke Programme Early CT score; CE-ASPECTS: contrast extravasation ASPECTS; ICH: intracranial haemorrhage; mRS: modified Rankin Scale; NIHSS: National Institutes of Health Stroke Scale.

**Table 3.** Effect estimates for the association between CE-ASPECTS and iodine concentration and stroke outcomes.

Outcome measures	EE	CE-ASPECTS		Iodine concentration		Iodine concentration relative to SSS	
		Unadjusted (95% CI)	Adjusted (95% CI)	Unadjusted (95% CI)	Adjusted (95% CI)	Unadjusted (95% CI)	Adjusted (95% CI)
mRS at 90 days	cOR	**1.14 (1.06–1.21)**	**1.10 (1.03–1.18)**	**1.22 (1.10–1.35)**	**1.18 (1.06–1.32)**	**1.13 (1.03–1.24)**	**1.13 (1.02–1.25)**
NIHSS at 24–48 h	β	**0.81 (0.49**–**1.13)**	**0.57 (0.29–0.84)**	**1.16 (0.73–1.60)**	**0.68 (0.30–1.06)**	**0.89 (0.46–1.32)**	**0.48 (0.12–0.84)**
mRS 3–6 at 90 days	OR	**1.14 (1.06–1.23)**	1.09 (0.99–1.20)	**1.32 (1.10–1.58)**	**1.22 (1.02–1.46)**	**1.24 (1.03–1.50)**	**1.27 (1.02–1.58)**
Mortality at 90 days	OR	**1.13 (1.04–1.22)**	**1.14 (1.02–1.26)**	**1.16 (1.04–1.30)**	**1.14 (1.00–1.31)**	**1.10 (1.00–1.22)**	1.11 (0.99–1.25)
Stroke progression	OR	**1.16 (1.06–1.27)**	**1.14 (1.03–1.26)**	1.03 (0.90–1.16)	0.99 (0.86–1.15)	1.01 (0.90–1.13)	0.98 (0.86–1.11)
ICH	OR	**1.20 (1.05–1.37)**	**1.21 (1.06–1.39)**	**1.40 (1.08–1.82)**	**1.37 (1.04–1.81)**	1.43 (0.99–2.07)	1.42 (0.97–2.08)
Symptomatic ICH	OR	**1.20 (1.00–1.42)**	1.15 (0.95–1.38)	**1.23 (1.07–1.41)**	**1.19 (1.02–1.38)**	**1.17 (1.04–1.32)**	1.13 (0.99–1.29)

ASPECTS: Alberta Stroke Programme Early CT score; CE-ASPECTS: contrast extravasation ASPECTS; EE: effect estimate; ICH: intracranial haemorrhage; mRS: modified Rankin Scale; NIHSS: National Institutes of Health Stroke Scale; SSS: superior sagittal sinus.

Adjustments: based on univariate analyses (Supplemental Appendix II).

Statistically significant estimates are bold.

Iodine concentration and iodine concentration relative to the SSS were associated with the mRS, NIHSS and dichotomised mRS (Table 3). For mortality at 90 days, only iodine concentration showed a significant association (Table 3).

### Association with safety outcomes

Safety outcomes per CE-ASPECTS category are shown in Table 2. Stroke progression occurred in 59/402 patients (15%). This was significantly associated with the CE-ASPECTS, but not with iodine concentration measures (Table 3).

One hundred forty patients received follow-up imaging, of which 35 demonstrated ICH (Supplemental Appendix III). Of all ICHs, 14 (40%) were symptomatic. The median time to ICH development was 55 h.

ICH development was significantly associated with the CE-ASPECTS and iodine concentration (Table 3). sICH was only associated with iodine concentration. Relative iodine concentration was not associated with (s)ICH (Table 3).

### Sensitivity analysis

We performed a sensitivity analysis in order to assess whether the exclusion of patients with asymptomatic ICH on DECT had an effect on our results. In this analysis, only patients with sICH on DECT (*n* = 18) were excluded, resulting in a sample size of 463. The sensitivity analysis yielded highly similar results (Supplemental Appendix V).

## Discussion

Our study demonstrates a clear association between immediate post-procedural CE on DECT and a wide range of stroke outcomes. This association was present for both the CE-ASPECTS and iodine concentration.

The CE-ASPECTS and iodine concentration were both predictive of the mRS at 90 days, NIHSS at 24–48 h, mortality at 90 days and ICH. These results are in accordance with previous literature, which demonstrated that post-EVT CE (vs no CE) is an independent predictor of the mRS at 90 days.^6^ One recent study also examined the association between the CE-ASPECTS and poor functional outcome at 90 days, mortality and ICH.^21^ However, as this study assessed CE on NCCT, it is likely that the inability to distinguish CE from ICH affected their results. Furthermore, the lack of information on the total amount of screened patients, combined with strict clinical in- and exclusion criteria, raises the question whether this cohort is representative for the larger stroke population. Our current analyses provide evidence for the applicability of the CE-ASPECTS in a broader population, and show the association of CE-ASPECTS with a wider range of outcomes (e.g. NIHSS at 24–48 h, mRS at 90 days and stroke progression).

Recently, a relationship between the volume of CE and ICH was described.^22^ Though this method may bear some resemblance to our CE-ASPECTS approach, differences in the included population hamper a direct comparison to our results. First, the previous study included a significant percentage of posterior circulation strokes. Second, only patients with follow-up imaging were considered, thereby selecting patients with a higher ICH risk. This is supported by the fact that their population had a median iodine concentration of 2.4 mg/ml (compared to 1.1 mg/ml in our population), and that 56% developed ICH (compared to 8.7% in our population).

In infarcted tissue, iodine concentrations may be higher due to more severe BBBD and possible stasis of contrast agent. Most previous studies on iodine concentration have therefore focussed on its relationship with ICH development.^8,23–25^ There are limitations, however, in comparing this previous work to our present study. Some studies included a large number of immediate post-procedural ICH in their analyses (35-62% of all ICHs), which limits their capability of describing the predictive value of iodine concentration on future ICH.^8,23,25^ Recently, a relationship between iodine concentration and ICH was described in a cohort of 56 patients, of which 50% developed ICH.^24^ The authors used a different approach in quantifying iodine concentration, namely the concentration of the 95th percentile in a volume of interest, and acknowledge that most ICHs in their study were small, possibly limiting clinical impact. In our current study, in which immediate post-procedural ICHs were excluded, iodine concentration was significantly related to ICH in multivariable regression. Thus, we were able to provide additional evidence that iodine concentration is an independent predictor of ICH.

In contrast to one previous study, our analyses did not show a significant relationship between ICH and relative iodine concentration.^9^ One possible explanation for this difference is the fact that the previous study only included patients with a pre-procedural ASPECTS ⩾7 in whom recanalisation was achieved. Furthermore, a considerable portion of their patient population without ICH on DECT demonstrated ICH on follow-up imaging (24%), which is substantially higher than the ICH rate in this study (8.7%). For the other outcome measures in our study, absolute and relative iodine concentration showed very similar effect sizes and model fits. Thus, we could not find convincing evidence for favouring relative over absolute iodine concentration.

Previous literature on the relationship between early CE and sICH is limited. One previous study suggested cortical CE as an independent predictor for sICH. However, given that they performed an NCCT instead of DECT, it is not possible to assess whether immediate post-procedural ICH had an impact on their results.^26^ Likewise, another study described a possible relationship between iodine concentration and sICH, but as 62% (*n* = 13) of these patients showed ICH on DECT, conclusions on the prediction of new sICH during follow-up cannot be drawn.^25^ In our current study, we only observed 14 sICHs on follow-up imaging. Hence, it is likely that our analyses for this outcome measure were underpowered. Nevertheless, iodine concentration was significantly associated with the occurrence of sICH. Our analyses between CE-ASPECTS and sICH did not reach statistical significance, though a trend towards significance may have been present.

### Generalisability and application

Our cohort is an adequate representation of the EVT-population in the Netherlands, as demonstrated by the high concordance with results from the MR CLEAN Registry.^15^ 79% of our cohort demonstrated early CE. This is in agreement with previous studies, and it supports our hypothesis that the mere distinction between CE absent versus present is not discriminative enough.^7–9,26,27^ We used the CE-ASPECTS instead, which was an independent predictor of all outcomes in this study, except for sICH. CE-ASPECTS was also the only CE-measure predictive of stroke progression. This corroborates our hypothesis that iodine concentration, which is measured in a small ROI, may not best reflect the total extent of ischaemic damage. Furthermore, though iodine concentration and CE-ASPECTS were both significantly related to several outcome measures, CE-ASPECTS may still be more discriminative. This is because CE-ASPECTS is scored on a 10-point scale, whereas the range of iodine concentration was far more limited in our cohort.

In this study, we scored CE-ASPECTS on IOM reconstructions. IOM reconstructions make it easier to appreciate subtle signs of CE, as brain parenchyma may appear isodense on mixed-energy images due to pseudo-normalisation.^28,29^ Still, it could be possible to score CE-ASPECTS on NCCT in centres which lack DECT capabilities, considering the aforementioned limitations of this application.

Routine CT follow-up after EVT is recommended by certain stroke guidelines.^30^ While DECT has already been shown to be superior to NCCT in ICH detection and differentiation from CE, our present study demonstrates that CE on DECT may also be used for prognostication.^4^ Future studies may aim to research the added value of DECT in early clinical decision-making and prognostication after EVT further.

### Strengths and limitations

As post-EVT DECT has been routinely performed in our centre for over a decade, we had the opportunity to assess an extensive patient cohort. Strengths of our study include access to prospective stroke records and imaging assessment by experienced neuroradiologists.

Nevertheless, certain limitations of our study need to be acknowledged. First, as only 140 patients received follow-up imaging after post-EVT DECT, it is likely that the prevalence of (s)ICH in our cohort was underestimated, which may have influenced our results. Furthermore, if patients were quickly discharged to referring hospitals, data on stroke progression or (s)ICH were scarce, possibly biasing our results. Future studies are therefore necessary to validate our findings. Nevertheless, the incidence of sICH in our cohort (3.6%), from which patients with immediate post-EVT ICH had already been excluded, was only slightly below the reported incidence of sICH in the major stroke trials (4.4%).^1^ It is also probable that the high level of experience with DECT in our centre resulted in a higher IRR than could be expected elsewhere. Lastly, this being a single-centre study, external validity should be further evaluated.

## Conclusions

Early CE after EVT is a relevant prognostic marker in AIS patients. Both CE-ASPECTS and iodine concentration are independent predictors of short- and long-term outcomes, though CE-ASPECTS is likely a better predictor for stroke progression. The straightforward applicability of CE-ASPECTS warrants its consideration as an early indicator for complications and (long-term) clinical outcomes.

## Supplemental Material

sj-docx-1-eso-10.1177_23969873231157901 – Supplemental material for Early post-endovascular treatment contrast extravasation on dual-energy CT is associated with clinical and radiological stroke outcomes: A 10-year single-centre experience Click here for additional data file. Supplemental material, sj-docx-1-eso-10.1177_23969873231157901 for Early post-endovascular treatment contrast extravasation on dual-energy CT is associated with clinical and radiological stroke outcomes: A 10-year single-centre experience by Florentina ME Pinckaers, Max MG Mentink, Hieronymus D Boogaarts, Wim H van Zwam, Robert J van Oostenbrugge and Alida A Postma in European Stroke Journal
