# Supplementary material for: Early post-endovascular treatment contrast extravasation on dual-energy CT is associated with clinical and radiological stroke outcomes: A 10-year single-centre experience
Source: Eur Stroke J. 2023 Feb 22;8(2):508–16. doi: 10.1177/23969873231157901 (PMC10334176; doi:10.1177/23969873231157901)
Supplement: sj-docx-1-eso-10.1177_23969873231157901 – Supplemental material for Early post-endovascular treatment contrast extravasation on dual-energy CT is associated with clinical and radiological stroke outcomes: A 10-year single-centre experience [file sj-docx-1-eso-10.1177_23969873231157901.docx]

Supplemental Appendix

Early Post-Endovascular Treatment Contrast Extravasation on Dual-Energy CT is Associated with Clinical and Radiological Stroke Outcomes: a Ten-Year Single-Centre Experience

Florentina M.E. Pinckaers, MD, Max M.G. Mentink, MD, Hieronymus D. Boogaarts, MD, PhD, Wim H. van Zwam, MD, PhD, Robert J. van Oostenbrugge, MD, PhD, Alida A. Postma, MD, PhD

Table of Contents

[Appendix I Blood-brain barrier dysfunction 2](#_Toc125035418)

[Appendix II Adjustments multivariable regression 3](#_Toc125035419)

[Appendix III ICH on follow-up imaging 4](#_Toc125035420)

[Appendix IV Complete model output of multivariable analysis per outcome variable 5](#_Toc125035421)

[Appendix V Sensitivity analysis 14](#_Toc125035422)

[Appendix VI Association between ultravist and iodine density 15](#_Toc125035423)

# Appendix I Blood-brain barrier dysfunction


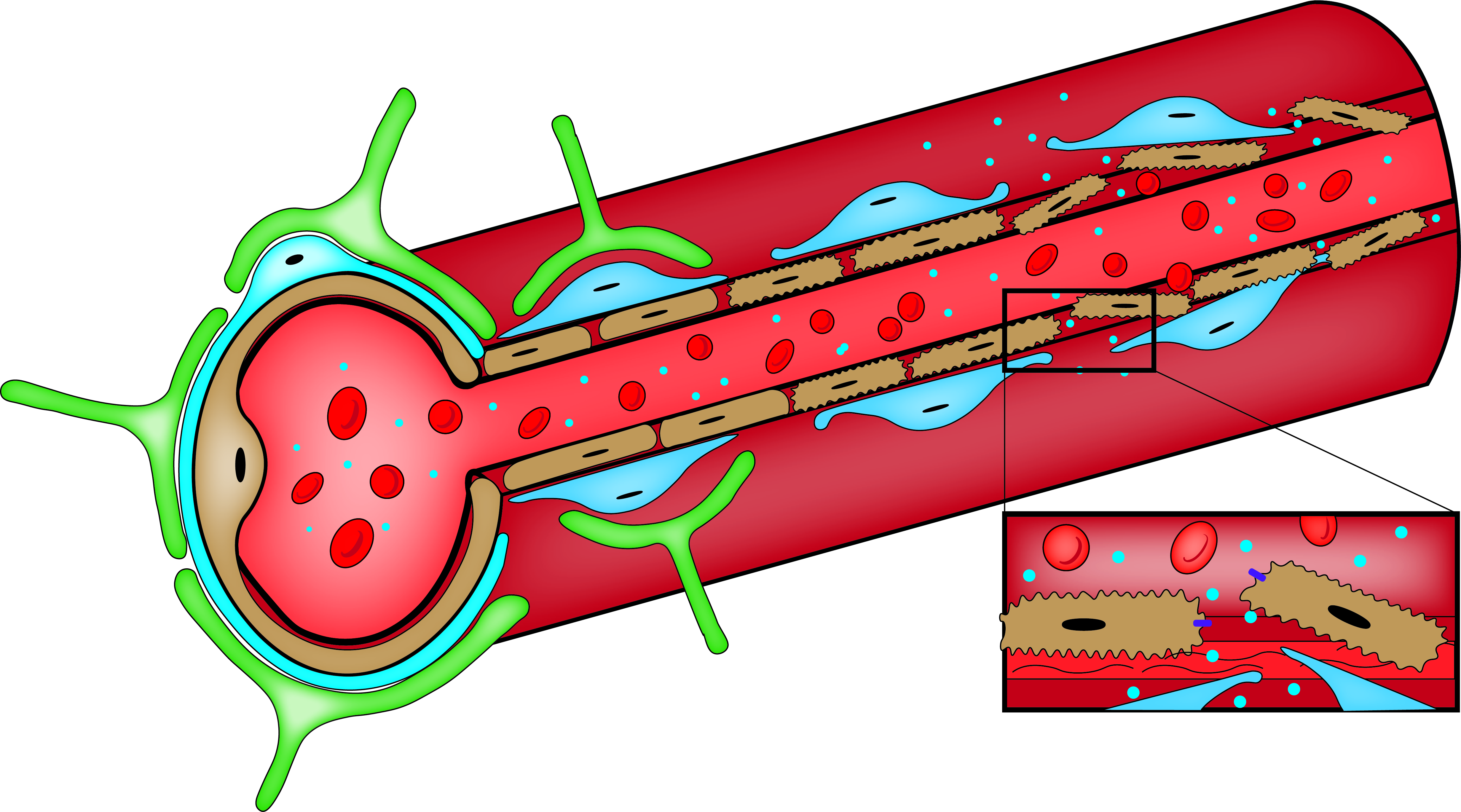


Figure S1: Schematic representation of contrast extravasation due to blood-brain barrier dysfunction (iodine molecules are denoted by blue dots).

# Appendix II Adjustments multivariable regression

Based on the results of univariate analyses, the following multivariable regression adjustments were made:

- *mRS at 90 days*: age, baseline NIHSS, intravenous alteplase, glucose, Hb, INR, history of diabetes mellitus, hypertension, atrial fibrillation, peripheral arterial disease, ICH, or ischemic stroke, pre-stroke dependence, usage of coumarins and antihypertensive drugs, occlusion segment, poor collaterals, EVT duration, recanalisation and total attempts;
- *NIHSS at 24-48 hours*: baseline NIHSS, glucose, INR, history of atrial fibrillation, usage of coumarins, occlusion segment, baseline ASPECTS, poor collaterals, EVT duration, recanalisation, total attempts and procedural complications;
- *mRS 3-6 vs. 0-2 at 90 days*: age, baseline NIHSS, intravenous alteplase, glucose, Hb, INR, history of atrial fibrillation or ischemic stroke, pre-stroke dependence, usage of coumarins and antihypertensive drugs, smoking, occlusion segment, baseline ASPECTS, poor collaterals, EVT duration, recanalisation and total attempts;
- *Death at 90 days*: age, baseline NIHSS, glucose, Hb, INR, history of diabetes mellitus, atrial fibrillation or peripheral arterial disease, pre-stroke dependence, occlusion segment, poor collaterals, EVT duration and recanalisation;
- *Stroke progression*: occlusion segment, poor collaterals, EVT duration, recanalisation and total attempts;
- *Any ICH (symptomatic or asymptomatic)*: glucose and poor collaterals.
- *Symptomatic ICH*: glucose and poor collaterals;

# Appendix III ICH on follow-up imaging

| Table S1: ICH prevalence on follow-up imaging | | |
| --- | --- | --- |
| **Type** | **Any ICH (n=35)** | **Symptomatic ICH (n=14)** |
| Hi1 | 7 | 0 |
| Hi2 | 6 | 0 |
| Ph1 | 9 | 4 |
| Ph2 | 10 | 10 |
| IVH | 9 | 7 |
| SAH | 8 | 4 |
| *Abbreviations*: ICH=intracranial haemorrhage. | | |

| Table S2: Frequencies of ICH combinations on follow-up imaging | |
| --- | --- |
| **Type** | **n** |
| **Heidelberg 1** | |
| HI1 | 7 |
| HI2 | 6 |
| PH1 | 5 |
| PH1 + SAH | 2 |
| PH1 + IVH | 2 |
| **Heidelberg 2** | |
| PH2 | 3 |
| PH2 + SAH | 2 |
| PH2 + IVH | 3 |
| PH2 + IVH + SAH | 2 |
| **Heidelberg 3** | |
| IVH | 1 |
| IVH + SAH | 1 |
| SAH | 1 |
| *Abbreviations*: HI=haemorrhagic infarction; PH=parenchymal haematoma; rPH=parenchymal haematoma remote from infarcted brain tissue; ICH=intracerbral haemorrhage; IVH=intraventricular haemorrhage; SAH=subarachnoid haemorrhage; SDH=subdural haemorrhage. | |

# Appendix IV Complete model output of multivariable analysis per outcome variable

| Table S3: Model output of ordinal regression analysis with the outcome measure mRS at 90 days | | | | | | | | | | | |
| --- | --- | --- | --- | --- | --- | --- | --- | --- | --- | --- | --- |
|  | **CE-ASPECTS** | | |  | **Iodine concentration** | | |  | **Iodine concentration relative to SSS** | | |
| **Independent variables** | **cOR** | **95%CI** | ***P*-value** | **Dd** | **cOR** | **95%CI** | ***P*-value** | **dd** | **cOR** | **95%CI** | ***P*-value** |
| Contrast extravasation* | 1.10 | 1.03 to 1.18 | **.01** |  | 1.18 | 1.06 to 1.32 | **.003** |  | 1.13 | 1.02 to 1.25 | **.02** |
| Age | 1.03 | 1.02 to 1.05 | **<.001** |  | 1.03 | 1.02 to 1.05 | **<.001** |  | 1.03 | 1.02 to 1.05 | **<.001** |
| Baseline NIHSS | 1.12 | 1.08 to 1.16 | **<.001** |  | 1.11 | 1.07 to 1.15 | **<.001** |  | 1.11 | 1.07 to 1.15 | **<.001** |
| IV-alteplase | 0.79 | 0.49 to 1.27 | .33 |  | 0.77 | 0.48 to 1.23 | .27 |  | 0.74 | 0.47 to 1.18 | .21 |
| Glucose | 1.09 | 1.00 to 1.17 | **.04** |  | 1.08 | 1.00 to 1.17 | .06 |  | 1.08 | 1.00 to 1.17 | .06 |
| Hb | 0.82 | 0.66 to 1.02 | .07 |  | 0.79 | 0.63 to 0.98 | **.03** |  | 0.79 | 0.64 to 0.99 | **.04** |
| INR | 1.75 | 0.65 to 4.71 | .27 |  | 1.58 | 0.59 to 4.25 | .36 |  | 1.63 | 0.61 to 4.36 | .33 |
| Diabetes Mellitus | 1.29 | 0.69 to 2.41 | .43 |  | 1.24 | 0.66 to 2.32 | .51 |  | 1.24 | 0.66 to 2.33 | .50 |
| Hypertension | 0.96 | 0.59 to 1.59 | .89 |  | 0.92 | 0.56 to 1.53 | .75 |  | 0.97 | 0.59 to 1.60 | .91 |
| Atrial fibrillation | 1.19 | 0.69 to 2.04 | .53 |  | 1.14 | 0.66 to 1.95 | .64 |  | 1.11 | 0.65 to 1.91 | .70 |
| Peripheral arterial disease | 2.29 | 1.08 to 4.82 | **.03** |  | 2.28 | 1.08 to 4.80 | **.03** |  | 2.34 | 1.11 to 4.94 | **.03** |
| Previous ICH | 5.36 | 0.81 to 35.45 | .08 |  | 5.90 | 0.89 to 39.14 | .07 |  | 5.36 | 0.81 to 35.45 | .08 |
| Previous ischemic stroke | 1.38 | 0.79 to 2.39 | .26 |  | 1.49 | 0.85 to 2.60 | .16 |  | 1.45 | 0.83 to 2.53 | .19 |
| Pre-stroke mRS >2 | 0.23 | 0.13 to 0.40 | **<.001** |  | 0.22 | 0.13 to 0.39 | **<.001** |  | 0.22 | 0.12 to 0.38 | **<.001** |
| Coumarin | 0.77 | 0.30 to 2.00 | .60 |  | 0.88 | 0.34 to 2.27 | .79 |  | 0.86 | 0.33 to 2.22 | .75 |
| Antihypertensive | 0.71 | 0.43 to 1.18 | .18 |  | 0.75 | 0.45 to 1.26 | .27 |  | 0.74 | 0.44 to 1.23 | .25 |
| ICA occlusion | 0.91 | 0.41 to 2.00 | .81 |  | 0.84 | 0.38 to 1.85 | .66 |  | 0.92 | 0.42 to 2.02 | .83 |
| ICA-T occlusion | 1.18 | 0.70 to 1.96 | .54 |  | 1.11 | 0.67 to 1.86 | .68 |  | 1.08 | 0.64 to 1.81 | .77 |
| M2 occlusion | 0.82 | 0.48 to 1.37 | .44 |  | 0.84 | 0.50 to 1.42 | .52 |  | 0.85 | 0.50 to 1.44 | .55 |
| Collaterals grade 2-3 | 0.47 | 0.31 to 0.72 | **<.001** |  | 0.45 | 0.29 to 0.69 | **<.001** |  | 0.45 | 0.29 to 0.69 | **<.001** |
| EVT duration | 1.01 | 1.01 to 1.02 | **<.001** |  | 1.01 | 1.01 to 1.02 | **<.001** |  | 1.01 | 1.01 to 1.02 | **<.001** |
| Recanalisation | 0.52 | 0.33 to 0.81 | **.004** |  | 0.53 | 0.34 to 0.84 | **.01** |  | 0.56 | 0.36 to 0.88 | **.01** |
| Total attempts | 0.98 | 0.87 to 1.11 | .77 |  | 0.99 | 0.88 to 1.12 | .87 |  | 1.01 | 0.90 to 1.14 | .82 |
| \| *Contrast extravasation denotes the estimates of either the CE-ASPECTS, iodine concentration or relative iodine concentration in the respective models.  *Abbreviations*: ASPECTS=Alberta Stroke Programme Early CT score; CE-ASPECTS=contrast extravasation ASPECTS; EVT=endovascular therapy; ICA=internal carotid artery; ICA-T=internal carotid artery terminus; ICH=intracranial haemorrhage; IV=intravenous; mRS=modified Rankin Scale; NIHSS=National Institutes of Health Stroke Scale; M2=middle cerebral artery; SSS=superior sagittal sinus.  **Note**: statistically significant estimates are bold. \| \| --- \| | | | | | | | | | | | |

| Table S4: Model output of linear regression analysis with the outcome measure NIHSS at 24-28 hours | | | | | | | | | | | |
| --- | --- | --- | --- | --- | --- | --- | --- | --- | --- | --- | --- |
|  | **CE-ASPECTS** | | |  | **Iodine concentration** | | |  | **Iodine concentration relative to SSS** | | |
| **Independent variables** | *β* | **95%CI** | ***P*-value** | **Dd** | *β* | **95%CI** | ***P*-value** | **dd** | *β* | **95%CI** | ***P*-value** |
| Contrast extravasation* | 0.57 | 0.29 to 0.84 | **<.001** |  | 0.68 | 0.30 to 1.06 | **<.001** |  | 0.48 | 0.12 to 0.84 | **.01** |
| Baseline NIHSS | 0.55 | 0.42 to 0.68 | **<.001** |  | 0.52 | 0.39 to 0.65 | **<.001** |  | 0.52 | 0.39 to 0.65 | **<.001** |
| Glucose | 0.33 | 0.05 to 0.62 | **.02** |  | 0.32 | 0.03 to 0.61 | **.03** |  | 0.32 | 0.03 to 0.62 | **.03** |
| INR | 1.95 | -1.80 to 5.70 | .31 |  | 1.44 | -2.32 to 5.21 | .45 |  | 1.75 | -2.03 to 5.53 | .36 |
| Atrial fibrillation | 2.41 | 0.26 to 4.57 | **.03** |  | 2.37 | 0.20 to 4.53 | **.03** |  | 2.35 | 0.17 to 4.53 | **.03** |
| Coumarin | -0.68 | -4.63 to 3.28 | .74 |  | -0.21 | -4.18 to 3.77 | .92 |  | -0.42 | -4.42 to 3.58 | .84 |
| ICA occlusion | 2.83 | -0.12 to 5.78 | .06 |  | 2.75 | -0.22 to 5.71 | .07 |  | 2.91 | -0.07 to 5.88 | .06 |
| ICA-T occlusion | 0.83 | -1.34 to 2.99 | .45 |  | 0.60 | -1.58 to 2.78 | .59 |  | 0.53 | -1.67 to 2.73 | .64 |
| M2 occlusion | 1.26 | -0.86 to 3.38 | .24 |  | 1.65 | -0.46 to 3.77 | .13 |  | 1.72 | -0.41 to 3.85 | .11 |
| Baseline ASPECTS | -0.25 | -0.68 to 0.19 | .27 |  | -0.33 | -0.76 to 0.10 | .14 |  | -0.35 | -0.78 to 0.08 | .11 |
| Collaterals grade 2-3 | -2.02 | -3.70 to -0.34 | **.02** |  | -2.23 | -3.92 to -0.54 | **.01** |  | -2.26 | -3.95 to -0.56 | **.01** |
| EVT duration | 0.05 | 0.03 to 0.08 | **<.001** |  | 0.06 | 0.03 to 0.08 | **<.001** |  | 0.06 | 0.03 to 0.08 | **<.001** |
| Recanalisation | -2.98 | -4.76 to -1.21 | **.001** |  | -2.78 | -4.57 to -1.00 | **.002** |  | -2.64 | -4.43 to -0.85 | **.004** |
| Total attempts | 0.12 | -0.36 to 0.61 | .62 |  | 0.19 | -0.29 to 0.68 | .44 |  | 0.28 | -0.21 to 0.76 | .27 |
| Procedural complications | 0.17 | -1.67 to 2.01 | .85 |  | 0.07 | -1.77 to 1.90 | .94 |  | 0.10 | -1.75 to 1.95 | .92 |
| \| *Contrast extravasation denotes the estimates of either the CE-ASPECTS, iodine concentration or relative iodine concentration in the respective models.  *Abbreviations*: ASPECTS=Alberta Stroke Programme Early CT score; CE-ASPECTS=contrast extravasation ASPECTS; EVT=endovascular therapy; ICA=internal carotid artery; ICA-T=internal carotid artery terminus; NIHSS=National Institutes of Health Stroke Scale; M2=middle cerebral artery; SSS=superior sagittal sinus.  **Note**: statistically significant estimates are bold. \| \| --- \| | | | | | | | | | | | |

| Table S5: Model output of binary logistic regression analysis with the outcome measure mRS 3-6 at 90 days | | | | | | | | | | | |
| --- | --- | --- | --- | --- | --- | --- | --- | --- | --- | --- | --- |
|  | **CE-ASPECTS** | | |  | **Iodine concentration** | | |  | **Iodine concentration relative to SSS** | | |
| **Independent variables** | **OR** | **95%CI** | ***P*-value** | **Dd** | **OR** | **95%CI** | ***P*-value** | **dd** | **OR** | **95%CI** | ***P*-value** |
| Contrast extravasation* | 1.09 | 0.99 to 1.20 | .09 |  | 1.22 | 1.02 to 1.46 | **.03** |  | 1.27 | 1.02 to 1.58 | **.03** |
| Age | 1.05 | 1.02 to 1.08 | **<.001** |  | 1.05 | 1.02 to 1.08 | **<.001** |  | 1.05 | 1.02 to 1.08 | **<.001** |
| Baseline NIHSS | 1.12 | 1.06 to 1.18 | **<.001** |  | 1.11 | 1.05 to 1.17 | **<.001** |  | 1.11 | 1.05 to 1.17 | **<.001** |
| IV-alteplase | 0.52 | 0.27 to 1.02 | .06 |  | 0.51 | 0.26 to 1.00 | .05 |  | 0.50 | 0.26 to 0.98 | **.04** |
| Glucose | 1.08 | 0.96 to 1.22 | .19 |  | 1.07 | 0.94 to 1.21 | .32 |  | 1.06 | 0.93 to 1.21 | .36 |
| Hb | 0.71 | 0.52 to 0.96 | **.03** |  | 0.69 | 0.50 to 0.93 | **.02** |  | 0.68 | 0.50 to 0.92 | **.01** |
| INR | 0.96 | 0.19 to 4.80 | .96 |  | 0.89 | 0.17 to 4.59 | .89 |  | 0.89 | 0.17 to 4.63 | .89 |
| Atrial fibrillation | 0.58 | 0.27 to 1.27 | .17 |  | 0.56 | 0.26 to 1.23 | .15 |  | 0.54 | 0.24 to 1.18 | .12 |
| Previous ischemic stroke | 2.07 | 0.86 to 5.01 | .10 |  | 2.21 | 0.92 to 5.31 | .08 |  | 2.19 | 0.91 to 5.27 | .08 |
| Pre-stroke mRS >2 | 0.06 | 0.02 to 0.22 | **<.001** |  | 0.06 | 0.01 to 0.22 | **<.001** |  | 0.05 | 0.01 to 0.21 | **<.001** |
| Coumarin | 3.42 | 0.70 to 16.60 | .13 |  | 3.94 | 0.81 to 19.24 | .09 |  | 3.90 | 0.79 to 19.29 | .09 |
| Antihypertensive | 0.82 | 0.46 to 1.46 | .49 |  | 0.86 | 0.48 to 1.53 | .60 |  | 0.88 | 0.49 to 1.57 | .66 |
| Smoking | 1.18 | 0.60 to 2.33 | .62 |  | 1.11 | 0.56 to 2.19 | .77 |  | 1.16 | 0.58 to 2.30 | .68 |
| ICA occlusion | 1.36 | 0.44 to 4.19 | .59 |  | 1.25 | 0.39 to 3.94 | .71 |  | 1.27 | 0.40 to 4.02 | .68 |
| ICA-T occlusion | 1.18 | 0.57 to 2.45 | .66 |  | 1.08 | 0.52 to 2.28 | .83 |  | 1.07 | 0.50 to 2.27 | .86 |
| M2 occlusion | 0.69 | 0.32 to 1.47 | .33 |  | 0.69 | 0.32 to 1.49 | .34 |  | 0.69 | 0.32 to 1.49 | .34 |
| Baseline ASPECTS | 0.93 | 0.80 to 1.09 | .38 |  | 0.92 | 0.79 to 1.08 | .31 |  | 0.92 | 0.79 to 1.08 | .30 |
| Collaterals grade 2-3 | 0.51 | 0.28 to 0.92 | **.03** |  | 0.49 | 0.27 to 0.89 | **.02** |  | 0.48 | 0.26 to 0.88 | **.02** |
| EVT duration | 1.02 | 1.01 to 1.03 | **.001** |  | 1.02 | 1.01 to 1.03 | **<.001** |  | 1.02 | 1.01 to 1.03 | **<.001** |
| Recanalisation | 0.48 | 0.25 to 0.90 | **.02** |  | 0.48 | 0.25 to 0.90 | **.02** |  | 0.48 | 0.25 to 0.91 | **.02** |
| Total attempts | 1.02 | 0.86 to 1.21 | .83 |  | 1.01 | 0.85 to 1.20 | .91 |  | 1.02 | 0.86 to 1.22 | .79 |
| \| *Contrast extravasation denotes the estimates of either the CE-ASPECTS, iodine concentration or relative iodine concentration in the respective models.  *Abbreviations*: ASPECTS=Alberta Stroke Programme Early CT score; CE-ASPECTS=contrast extravasation ASPECTS; EVT=endovascular therapy; ICA=internal carotid artery; ICA-T=internal carotid artery terminus; IV=intravenous; mRS=modified Rankin Scale; NIHSS=National Institutes of Health Stroke Scale; M2=middle cerebral artery; SSS=superior sagittal sinus.  **Note**: statistically significant estimates are bold. \| \| --- \| | | | | | | | | | | | |

| Table S6: Model output of binary logistic regression analysis with the outcome measure mortality at 90 days | | | | | | | | | | | |
| --- | --- | --- | --- | --- | --- | --- | --- | --- | --- | --- | --- |
|  | **CE-ASPECTS** | | |  | **Iodine concentration** | | |  | **Iodine concentration relative to SSS** | | |
| **Independent variables** | **OR** | **95%CI** | ***P*-value** | **Dd** | **OR** | **95%CI** | ***P*-value** | **dd** | **OR** | **95%CI** | ***P*-value** |
| Contrast extravasation* | 1.14 | 1.02 to 1.26 | **.02** |  | 1.14 | 1.00 to 1.31 | **.05** |  | 1.11 | 0.99 to 1.25 | .07 |
| Age | 1.06 | 1.03 to 1.09 | **<.001** |  | 1.06 | 1.03 to 1.09 | **<.001** |  | 1.06 | 1.03 to 1.09 | **<.001** |
| Baseline NIHSS | 1.12 | 1.06 to 1.18 | **<.001** |  | 1.11 | 1.05 to 1.17 | **<.001** |  | 1.11 | 1.05 to 1.17 | **<.001** |
| Glucose | 1.08 | 0.97 to 1.19 | .16 |  | 1.08 | 0.97 to 1.20 | .17 |  | 1.08 | 0.97 to 1.20 | .18 |
| Hb | 0.81 | 0.58 to 1.12 | .20 |  | 0.77 | 0.55 to 1.07 | .12 |  | 0.78 | 0.56 to 1.08 | .14 |
| INR | 1.67 | 0.62 to 4.49 | .31 |  | 1.46 | 0.55 to 3.92 | .45 |  | 1.53 | 0.58 to 4.08 | .39 |
| Diabetes Mellitus | 1.68 | 0.71 to 3.94 | .23 |  | 1.58 | 0.67 to 3.74 | .29 |  | 1.58 | 0.67 to 3.71 | .29 |
| Atrial fibrillation | 1.47 | 0.71 to 3.04 | .30 |  | 1.45 | 0.70 to 3.02 | .31 |  | 1.42 | 0.68 to 2.93 | .35 |
| Peripheral arterial disease | 3.16 | 1.22 to 8.19 | **.02** |  | 3.12 | 1.21 to 8.03 | **.02** |  | 3.17 | 1.24 to 8.10 | **.02** |
| Pre-stroke mRS >2 | 0.36 | 0.18 to 0.73 | **.005** |  | 0.35 | 0.17 to 0.72 | **.004** |  | 0.34 | 0.17 to 0.70 | **.003** |
| ICA occlusion | 1.90 | 0.63 to 5.75 | .25 |  | 1.89 | 0.63 to 5.63 | .25 |  | 1.97 | 0.66 to 5.82 | .22 |
| ICA-T occlusion | 1.72 | 0.79 to 3.72 | .17 |  | 1.65 | 0.76 to 3.59 | .20 |  | 1.60 | 0.74 to 3.48 | .23 |
| M2 occlusion | 1.04 | 0.45 to 2.39 | .93 |  | 1.12 | 0.49 to 2.57 | .79 |  | 1.13 | 0.49 to 2.58 | .77 |
| Collaterals grade 2-3 | 0.47 | 0.25 to 0.88 | **.02** |  | 0.43 | 0.23 to 0.81 | **.01** |  | 0.43 | 0.23 to 0.81 | **.01** |
| EVT duration | 1.01 | 1.00 to 1.02 | **.05** |  | 1.01 | 1.00 to 1.02 | **.04** |  | 1.01 | 1.00 to 1.02 | **.03** |
| Recanalisation | 0.39 | 0.20 to 0.74 | **.005** |  | 0.43 | 0.23 to 0.82 | **.01** |  | 0.45 | 0.24 to 0.86 | **.02** |
| \| *Contrast extravasation denotes the estimates of either the CE-ASPECTS, iodine concentration or relative iodine concentration in the respective models.  Abbreviations: ASPECTS=Alberta Stroke Programme Early CT score; CE-ASPECTS=contrast extravasation ASPECTS; EVT=endovascular therapy; ICA=internal carotid artery; ICA-T=internal carotid artery terminus; IV=intravenous; mRS=modified Rankin Scale; NIHSS=National Institutes of Health Stroke Scale; M2=middle cerebral artery; SSS=superior sagittal sinus.  **Note**: statistically significant estimates are bold. \| \| --- \| | | | | | | | | | | | |

| Table S7: Model output of binary logistic regression analysis with the outcome measure stroke progression | | | | | | | | | | | |
| --- | --- | --- | --- | --- | --- | --- | --- | --- | --- | --- | --- |
|  | **CE-ASPECTS** | | |  | **Iodine concentration** | | |  | **Iodine concentration relative to SSS** | | |
| **Independent variables** | **OR** | **95%CI** | ***P*-value** | **Dd** | **OR** | **95%CI** | ***P*-value** | **dd** | **OR** | **95%CI** | ***P*-value** |
| Contrast extravasation* | 1.14 | 1.03 to 1.26 | **.01** |  | 0.99 | 0.86 to 1.15 | .93 |  | 0.98 | 0.86 to 1.11 | .71 |
| ICA occlusion | 0.80 | 0.26 to 2.45 | .69 |  | 0.84 | 0.28 to 2.56 | .76 |  | 0.84 | 0.28 to 2.56 | .76 |
| ICA-T occlusion | 0.79 | 0.34 to 1.86 | .59 |  | 0.78 | 0.33 to 1.85 | .57 |  | 0.79 | 0.33 to 1.87 | .59 |
| M2 occlusion | 1.63 | 0.78 to 3.42 | .20 |  | 1.77 | 0.85 to 3.67 | .13 |  | 1.76 | 0.84 to 3.66 | .13 |
| Collaterals grade 2-3 | 0.55 | 0.30 to 1.02 | .06 |  | 0.53 | 0.29 to 0.98 | **.04** |  | 0.53 | 0.29 to 0.97 | **.04** |
| EVT duration | 1.02 | 1.01 to 1.03 | **<.001** |  | 1.02 | 1.01 to 1.03 | **<.001** |  | 1.02 | 1.01 to 1.03 | **<.001** |
| Recanalisation | 0.44 | 0.24 to 0.81 | **.01** |  | 0.48 | 0.26 to 0.89 | **.02** |  | 0.48 | 0.26 to 0.88 | **.02** |
| Total attempts | 1.00 | 0.84 to 1.18 | .97 |  | 1.03 | 0.87 to 1.21 | .74 |  | 1.03 | 0.87 to 1.21 | .74 |
| \| *Contrast extravasation denotes the estimates of either the CE-ASPECTS, iodine concentration or relative iodine concentration in the respective models.  Abbreviations: ASPECTS=Alberta Stroke Programme Early CT score; CE-ASPECTS=contrast extravasation ASPECTS; EVT=endovascular therapy; ICA=internal carotid artery; ICA-T=internal carotid artery terminus; M2=middle cerebral artery; SSS=superior sagittal sinus.  **Note**: statistically significant estimates are bold. \| \| --- \| | | | | | | | | | | | |

| Table S8: Model output of binary logistic regression analysis with the outcome measure ICH | | | | | | | | | | | |
| --- | --- | --- | --- | --- | --- | --- | --- | --- | --- | --- | --- |
|  | **CE-ASPECTS** | | |  | **Iodine concentration** | | |  | **Iodine concentration relative to SSS** | | |
| **Independent variables** | **OR** | **95%CI** | ***P*-value** | **Dd** | **OR** | **95%CI** | ***P*-value** | **dd** | **OR** | **95%CI** | ***P*-value** |
| Contrast extravasation* | 1.21 | 1.06 to 1.39 | **.01** |  | 1.37 | 1.04 to 1.81 | **.03** |  | 1.42 | 0.97 to 2.08 | .07 |
| Glucose | 1.18 | 0.99 to 1.41 | .07 |  | 1.12 | 0.94 to 1.35 | .21 |  | 1.13 | 0.94 to 1.36 | .20 |
| Collaterals grade 2-3 | 1.02 | 0.44 to 2.39 | .96 |  | 1.10 | 0.46 to 2.64 | .83 |  | 1.07 | 0.44 to 2.56 | .88 |
| \| *Contrast extravasation denotes the estimates of either the CE-ASPECTS, iodine concentration or relative iodine concentration in the respective models. \| \| --- \| \| Abbreviations: ASPECTS=Alberta Stroke Programme Early CT score; CE-ASPECTS=contrast extravasation ASPECTS; SSS=superior sagittal sinus. \| \| **Note**: statistically significant estimates are bold. \| | | | | | | | | | | | |

| Table S9: Model output of binary logistic regression analysis with the outcome measure symptomatic ICH | | | | | | | | | | | |
| --- | --- | --- | --- | --- | --- | --- | --- | --- | --- | --- | --- |
|  | **CE-ASPECTS** | | |  | **Iodine concentration** | | |  | **Iodine concentration relative to SSS** | | |
| **Independent variables** | **OR** | **95%CI** | ***P*-value** | **Dd** | **OR** | **95%CI** | ***P*-value** | **dd** | **OR** | **95%CI** | ***P*-value** |
| Contrast extravasation* | 1.15 | 0.95 to 1.38 | .15 |  | 1.19 | 1.02 to 1.38 | **.02** |  | 1.13 | 0.99 to 1.29 | .06 |
| Glucose | 1.12 | 0.99 to 1.26 | .07 |  | 1.10 | 0.97 to 1.25 | .13 |  | 1.10 | 0.96 to 1.26 | .17 |
| Collaterals grade 2-3 | 0.30 | 0.09 to 1.00 | .05 |  | 0.30 | 0.09 to 1.02 | .05 |  | 0.29 | 0.09 to 0.99 | **.05** |
| \| *Contrast extravasation denotes the estimates of either the CE-ASPECTS, iodine concentration or relative iodine concentration in the respective models.  Abbreviations: ASPECTS=Alberta Stroke Programme Early CT score; CE-ASPECTS=contrast extravasation ASPECTS; SSS=superior sagittal sinus.  **Note**: statistically significant estimates are bold. \| \| --- \| | | | | | | | | | | | |

# Appendix V Sensitivity analysis

In the sensitivity analysis, only patients with symptomatic ICH on DECT were excluded (as opposed to any ICH). Adjustments were kept the same as in the main analysis.

| Table S10: Effect estimates for the association between CE-ASPECTS & iodine concentration and clinical & radiological outcomes | | | | | | | |
| --- | --- | --- | --- | --- | --- | --- | --- |
|  |  | **CE-ASPECTS** | | **Iodine concentration** | | **Iodine concentration relative to SSS** | |
| **Outcome measures** | **EE** | **Unadjusted (95%CI)** | **Adjusted (95%CI)** | **Unadjusted (95%CI)** | **Adjusted (95%CI)** | **Unadjusted (95%CI)** | **Adjusted (95%CI)** |
| mRS at 90 days | cOR | **1.15 (1.08-1.22)** | **1.11 (1.04-1.18)** | **1.23 (1.12-1.37)** | **1.19 (1.06-1.33)** | **1.15 (1.05-1.27)** | **1.15 (1.03-1.28)** |
| NIHSS at 24-28h | *β* | **0.79 (0.49-1.09)** | **0.50 (0.23-0.77)** | **1.06 (0.66-1.46)** | **0.57 (0.20-0.93)** | **0.91 (0.50-1.33)** | **0.51 (0.14-0.87)** |
| mRS 3-6 at 90 days | OR | **1.15 (1.07-1.23)** | **1.10 (1.00-1.20)** | **1.28 (1.08-1.52)** | 1.19 (1.00-1.43) | **1.22 (1.03-1.45)** | **1.25 (1.01-1.55)** |
| Mortality at 90 days | OR | **1.15 (1.06-1.23)** | **1.15 (1.04-1.26)** | **1.19 (1.08-1.33)** | **1.15 (1.01-1.31)** | **1.13 (1.03-1.25)** | 1.12 (1.00-1.27) |
| Stroke progression | OR | **1.15 (1.05-1.25)** | **1.12 (1.02-1.24)** | 1.03 (0.93-1.15) | 1.02 (0.90-1.14) | 1.00 (0.90-1.12) | 0.98 (0.87-1.10) |
| ICH | OR | **1.22 (1.08-1.38)** | **1.23 (1.09-1.39)** | **1.43 (1.08-1.90)** | **1.42 (1.06-1.90)** | 1.39 (0.99-1.93) | 1.38 (0.98-1.96) |
| Symptomatic ICH | OR | **1.17 (1.01-1.35)** | 1.13 (0.98-1.31) | **1.17 (1.04-1.31)** | **1.14 (1.01-1.29)** | **1.14 (1.02-1.27)** | 1.11 (0.99-1.25) |
| *Abbreviations*: ASPECTS=Alberta Stroke Programme Early CT score; CE-ASPECTS=contrast extravasation ASPECTS; EE=effect estimate; ICH=intracranial haemorrhage; mRS=modified Rankin Scale; NIHSS=National Institutes of Health Stroke Scale; SSS=superior sagittal sinus.  *Adjustments*: based on univariate analyses. See Supplemental Appendix II for details.  **Note**: statistically significant estimates are bold. | | | | | | | |

# Appendix VI Association between ultravist and iodine density

A

B


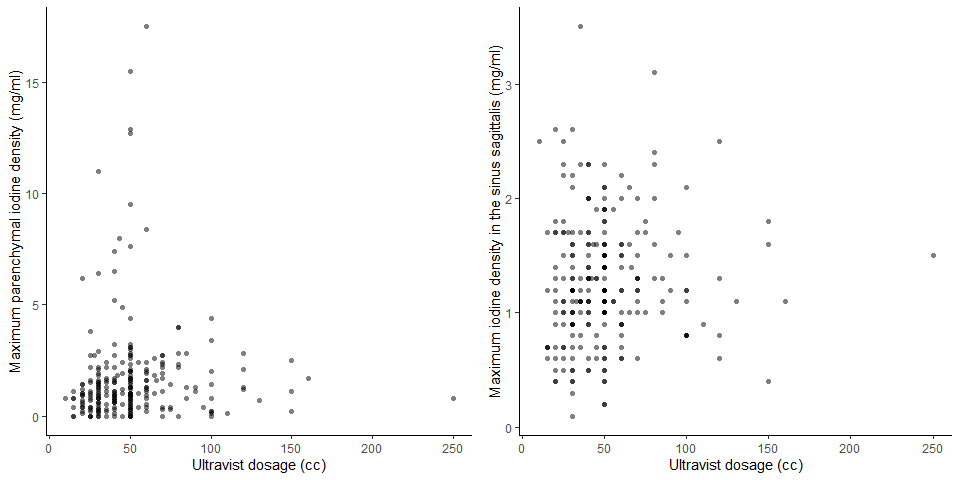


A

B

*Figure S2: Relationship between the ultravist dosage, and the maximum parenchymal iodine density (A) and maximum iodine density in the sinus sagittalis (B).*

| Table S11: Correlation between ultravist dosage (cc) and iodine concentration measures (mg/ml) | | |
| --- | --- | --- |
|  | **Parenchymal iodine densitiy** | **Sinus sagittalis iodine density** |
| *P*-value | **.003** | .11 |
| Rho | 0.19 | 0.10 |
